# The Efficacy of Citroline in the Treatment of Ischemic Stroke and Primary Hypertensive Intracereral Hemorrhage; A Review Article

**Published:** 2010

**Authors:** Seyed Ali Mousavi, Fariborz Khorvash, Tahereh Hoseini

**Affiliations:** 1 MD, Associate Professor of Neurology, Department of Neurology, Alzahra Hospital, Isfahan University of Medical Sciences, Isfahan, Iran; 2 MD, Assistant Professor of Neurology, Department of Neurology, Alzahra Hospital, Isfahan University of Medical Sciences, Isfahan, Iran; 3 Department of Neurology, Alzahra Hospital, Isfahan University of Medical Sciences, Isfahan, Iran

**Keywords:** Citicoline, Treatment, Ischemic Stroke, Intracerebral Hemorrhage

## Abstract

Stroke is a medical emergency with a mortality rate higher than most forms of cancer. It is the second leading cause of death in developed countries and the most common cause of serious, long-term disability in adults.

Primary intracerebral hemorrhage (ICH) is a major clinical problem that accounts for 15% of all acute stroke hospitalizations. Currently, there is no medical therapy available for these patients, with options being limited to supportive care or invasive neurosurgical evacuation. The damage induced by an ICH appears to be related to a combination of different factors. In addition to direct mechanical disruption from the hematoma, surrounding injury secondary to edema formation and ischemia are contributing factors for brain injury following ICH. Citicoline (cytidine-5-diphosphocholine) is an essential precursor for the synthesis of phosphatidylcholine that is key component of cell membranes. Citicoline is a naturally occurring endogenous compound. For clinical use, the sodium salt of this compound usually utilized. During ischemia, phosphatidylcholine is broken down into free fatty acids, which in turn are used to generate free radicals that potentiate ischemic injury. Citicoline is a neuroprotectant drug with some beneficial effects in human ischemic stroke and primary intracerebral hemorrhage (ICH) with an excellent safety profile.

In the current paper, we review published papers regarding use of citicoline in the treatment of human ischemic stroke and primary intracerebral hemorrhage (ICH).

## Introduction

Stroke is a medical emergency with a mortality rate higher than most forms of cancer. It is the second leading cause of death in developed countries and the most common cause of serious, long-term disability in adults.^1^

Primary intracerebral hemorrhage (ICH) is a major clinical problem that accounts for 15% of all acute stroke hospitalizations.^2^ Mousavi et al. mentioned Primary Hypertensive ICH and recurrence of that is an importance health problem.^3^ Currently, there is no medical therapy available for these patients, with options being limited to supportive care or invasive neurosurgical evacuation. Citicoline (CDP-choline, cytidine diphosphate choline or cytidine 5’-diphosphocholine) belongs to the group of biomolecules in living systems recognized as “nucleotides” that have important roles in cellular metabolism.^4^

Grouped with the B vitamins, choline is a trimethylated nitrogenous base that goes into three major metabolic pathways: (1) phospholipid synthesis via phosphorylcholine; (2) acetylcholine synthesis; and (3) oxidation to betaine, which serves as a methyl donor.^5^

It is recognized that choline enters the various biosynthetic pathways that use citicoline as an intermediate. Citicoline, therefore, has a sparing effect on systemic choline reserves, as well as inhibiting the breakdown of membrane phospholipids.^6^

Citicoline is a water-soluble compound with greater than 90-percent bioavailability and is metabolized in the gut wall and liver.^7^

Studies have shown that citicoline elimination occurs mainly through respiratory

CO2 and urinary excretion. The elimination half-life is 56 hours for CO2 and 71 hours for urinary excretion.^7^

Four different mechanisms of action have been suggested for citicoline that include;

1. They have a role as a phosphatidylcholine precursor (as has been shown in animal studies)^8^ and therefore exogenous citicoline helps preserve the structural and functional integrity of the neuronal membrane.
2. They have a role in repair of neuronal membrane that makes them as a candidate for stroke therapy. The main mechanisms in this regards are;
  Repair of neuronal membranes via increased synthesis of phosphatidylcholine.
  Repair of damaged cholinergic neurons via potentiation of acetylcholine production.
  Reduction of free fatty acid buildup at the site of stroke-induced nerve damage.^9^
3. Citicoline counteracts the deposition of beta-amyloid, a neurotoxic protein, which believed to play a central role in the pathophysiology of Alzheimer's disease.^7^
4. In human, it has been shown that citicoline can enhance norepinephrine release. Citicoline potentiates dopamine release in the brain of rats, presumably by stimulating release of acetylcholine.^7^

Post-stroke rehabilitation, ischemic stroke, hemorrhagic stroke, dementia, Alzheimer's disease, brain trauma, spinal cord injury, Parkinson's disease, Huntington's disease, bipolar disorder and associated substance abuse and finally glaucoma and amblyopia are regarded as clinical indications of citicoline.

### Efficacy of citicoline as an acute stroke treatment

Numerous experimental studies with citicoline have shown improved outcome and reduced infarct size in ischemic stroke models .^10^ Citicoline has been studied worldwide in both ischemic clinical stroke with excellent safety and possibly efficacy found in several trials).^11^

Efficacy of 3 different doses of citicoline (i.e. 500 mg, 1000 mg and 2000 mg) in

patients with acute ischemic stroke were evaluated by Clark et al.^12^ as a vehicle controlled double-blind trial. Treatment was initiated within 24 hours of stroke onset and was continued orally for 6 weeks. No drug-related serious adverse events or deaths were reported in this study. The authors concluded that oral citicoline could be used safely with minimal side effects in acute stroke treatment.

Clark et al.^13^ evaluated efficacy of citicoline (500 mg po daily) for 6 weeks, with a 6-week post treatment follow-up period in the patients with acute ischemic stroke as a randomized, double-blind, placebo-controlled clinical trial. Only patients with acute ischemic strokes in the middle cerebral artery territory with NIHSS > or = 5 were enrolled. Their results indicated that citicoline was safe, but ineffective in improving the outcome of patients with acute ischemic stroke who were enrolled in this trial. However, the authors concluded that citicoline might be useful in patients with moderate strokes.

In another randomized, double-blind trial, 899 patients with acute stroke were randomized to be treated either with placebo or with citicoline (1000 mg po twice a day) for 6 weeks, with a 6-week post-treatment follow up period. Patients with acute (< or =24 hours) ischemic strokes clinically thought to be in the middle cerebral artery territory with NIH Stroke Scale (NIHSS) scores > or =8 were evaluated. The authors concluded that citicoline was safe, but ineffective in improving the outcome of patients with acute ischemic stroke as measured by the planned analyses.^14^

A systematic search of all prospective, randomized, placebo-controlled, double-blind clinical trials with oral citicoline was also performed by Davalose et al.^4^

They reviewed four clinical trials using different doses of oral citicoline (i.e. 500, 1000, and 2000 mg). Out of 1652 randomized patients, 1372 fulfilled the inclusion criteria (583 received placebo, 789 received citicoline). Recovery at 3 months was 25.2% in citicoline-treated patients and 20.2% in placebo treated patients (P = 0.0034). 2000 mg of citicoline showed the largest difference with placebo with 27.9% of patients achieving recovery (P = 0.0043).

No significant difference regarding side effect was observed between the two groups. The authors concluded that treatment with oral citicoline within the first 24 hours after onset in patients with moderate to severe stroke increases the probability of complete recovery at three months period.^15^

Mousavi et al. selected patients who had cortical infarction in the middle cerebral artery territory (superior or inferior division) with moderate neurologic deficits (Orgogozo scale score greater than 30 and less than 70) and onset less than 24 hours. The patients were treated with citicoline (500 mg bid) for seven days and examined by a blind investigator. NIH stroke scale and orgogozo index were obtained on admission and compared with Barthel index and Rankin score in 90 days after stroke. 20 patients were treated and 20 patients were left untreated as controls. Significant difference in Barthel index was observed 90 days after stroke between two groups. The authors concluded that intravenous citicoline had significant beneficial effect on acute phase of stroke patients and this compound might be helpful for reduction of morbidity and disability of the stroke patients.^16^

In experimental stroke models, citicoline conferred acute neuroprotection and enhanced neuroplasticity and neurorepair in the subacute period. Although individual human stroke trials have been inconclusive, meta-analysis of 10 trials enrolling 2279 patients suggests patients receiving citicoline had substantially reduced frequencies of death and disability.^11^

Considering the aforementioned studies regarding efficacy of citicoline and its safety profile, it seems logical to use this medication in patients with acute phase of stroke.

### Efficacy of citicoline as a hemorrhagic stroke treatment

There are only few studies regarding use of citicoline for patients with ICH. Clark et al.^2^ evaluated the efficacy of citicoline in reduction of ischemic injury and improvement of the functional neurological outcome in an experimental model of ICH. ICH was induced by collagenase injection into the caudate nucleus in 68 Swiss albino mice. These mice were randomized to receive either citicoline 500 mg/kg or saline IP prior to collagenase at 24 and 48 hours. Mice were rated on a 28-point neurological scale and sacrificed at 54 hours. The brains were sectioned, and the volume of hematoma, total lesion, and surrounding ischemic injury was determined. Regarding functional outcome, animals treated with citicoline had improved neurological outcome scores compared with placebo-treated animals (P < 0.01). In terms of ischemic injury, animals treated with citicoline had a smaller surrounding volume of ischemic injury than placebo-treated animals (P < 0.05). However, there was no difference in the underlying hematoma volumes.

The authors concluded that treatment with citicoline significantly improved functional outcome and reduced the volume of ischemic injury surrounding the hematoma^2^.

Safety and efficacy of citicoline in human with intracerebral haemorrhage was evaluated by Secades et al.^17^ as a pilot study. In this double-blind, placebo-controlled study, patients with acute primary supratentorial hemispheric cerebral haemorrhage were selected and received either a placebo or 1 g/12 h citicoline for 2 weeks (orally or intravenously). The efficacy endpoint was the percentage of patients with a modified Rankin Score (mRS) at 3 months. The incidence of serious adverse events was not different among groups (4 patients in each group). One patient in the placebo group was categorized as independent (mRS < or = 2) in comparison with 5 patients in the citicoline group (OR, 5.38; 95% CI, 0.55-52). The authors concluded that citicoline was a safe drug in human intracerebral hemorrhage with a positive trend regarding efficacy .^17^

Mousavi et al. evaluated 40 patients aged older than 40-years old with acute symptoms of supratentorial hemorrhage and onset of symptoms less than 6 hours. Cases group were treated with citicoline (1000 mg bid) for 14 days and control group were treated with placebo.

At the end of study (at day 90), patients were examined again for NIHSS, GCS s and Barthel index. There was significant difference regarding Barthel index between the two groups in favor of citicoline group (p = 0.008). The authors concluded that citicoline can improve neuralgic function and can decrease symptoms of ICH.Very few patients showed side effects following treatment with citicoline .^18^

Overall, it seems that still there are not enough evidences to use citicoline in the treatment of human intracerebral hemorrhage. More randomized clinical trials with larger sample size are required to confirm the efficacy and safety of this medication for patients with ICH.
